# Identification of genetic biomarkers, drug targets and agents for respiratory diseases utilising integrated bioinformatics approaches

**DOI:** 10.1038/s41598-023-46455-8

**Published:** 2023-11-04

**Authors:** Fee Faysal Ahmed, Arnob Dip Das, Mst. Joynab Sumi, Md. Zohurul Islam, Md. Shahedur Rahman, Md. Harun Rashid, Salem A. Alyami, Naif Alotaibi, A. K. M. Azad, Mohammad Ali Moni

**Affiliations:** 1 Department of Mathematics, Faculty of Science, Jashore University of Science and Technology, Jashore, 7408 Bangladesh; 2 High Performance Computing (HPC) Laboratory, Department of Mathematics, Jashore University of Science and Technology, Jashore, 7408 Bangladesh; 3 Department of Genetic Engineering and Biotechnology, Jashore University of Science and Technology, Jashore, 7408 Bangladesh; 4 Bioinformatics and Microbial Biotechnology Laboratory, Department of Genetic Engineering and Biotechnology, Jashore University of Science and Technology, Jashore, 7408 Bangladesh; 5 Department of Mathematics and Statistics, College of Science, Imam Mohammad Ibn Saud Islamic University (IMSIU), 13318 Riyadh, Saudi Arabia; 6 Artificial Intelligence and Data Science, School of Health and Rehabilitation Sciences, Faculty of Health and Behavioural Sciences, The University of Queensland, St Lucia, QLD 4072 Australia

**Keywords:** Computational biology and bioinformatics, Molecular biology

## Abstract

Respiratory diseases (RD) are significant public health burdens and malignant diseases worldwide. However, the RD-related biological information and interconnection still need to be better understood. Thus, this study aims to detect common differential genes and potential hub genes (HubGs), emphasizing their actions, signaling pathways, regulatory biomarkers for diagnosing RD and candidate drugs for treating RD. In this paper we used integrated bioinformatics approaches (such as, gene ontology (GO) and KEGG pathway enrichment analysis, molecular docking, molecular dynamic simulation and network-based molecular interaction analysis). We discovered 73 common DEGs (CDEGs) and ten HubGs (*ATAD2B, PPP1CB, FOXO1, AKT3, BCR, PDE4D, ITGB1, PCBP2, CD44* and *SMARCA2*). Several significant functions and signaling pathways were strongly related to RD. We recognized six transcription factor (TF) proteins *(FOXC1, GATA2, FOXL1, YY1, POU2F2 and HINFP*) and five microRNAs (hsa-mir-218-5p, hsa-mir-335-5p, hsa-mir-16-5p, hsa-mir-106b-5p and hsa-mir-15b-5p) as the important transcription and post-transcription regulators of RD. Ten HubGs and six major TF proteins were considered drug-specific receptors. Their binding energy analysis study was carried out with the 63 drug agents detected from network analysis. Finally, the five complexes (the *PDE4D*-benzo[a]pyrene, *SMARCA2*-benzo[a]pyrene, *HINFP*-benzo[a]pyrene, *CD44*-ketotifen and *ATAD2B*-ponatinib) were selected for RD based on their strong binding affinity scores and stable performance as the most probable repurposable protein-drug complexes. We believe our findings will give readers, wet-lab scientists, and pharmaceuticals a thorough grasp of the biology behind RD.

## Introduction

Around a million people die each year from respiratory diseases (RD), which are the most prevalent cause of disease-related mortality globally^1,2^. Chronic obstructive pulmonary disease (COPD), influenza, pneumonia, emphysema, lung cancers, asthma and covid-19 are the most common RD^3,4^. Among these lung diseases, severe acute respiratory syndrome coronavirus (SARS-CoV-2, commonly known as COVID-19) is the very recent and world-threatening outbreak^5^.

New therapeutic or preventive methods that are common for these lung diseases need to be proposed. To uncover novel therapeutics for patients with lung diseases, we need a complete understanding of their biological or molecular mechanisms and interconnected biological information among the RD. However, the biological or molecular information, as well as drug or non-drug mechanisms of lung diseases, are yet to be not fully understood by existing literature. But, a better understanding of the mechanisms of these diseases is required for diagnosing RD and finding novel drugs. The gene expression profiles, biomarker exploration and bio-functional pathways could be the novel method to get information about the identification of possible treatment strategies. Wet lab equipment and the use of this sophisticated equipment could be very costly. More experienced operators, periodic maintenance, longer start-up time and huge space requirements are the major problems of wet lab experiments. To address these issues, bioinformatics approaches are becoming increasingly tool-to-go in biomedical research day by day. However, high-volume bioinformatics data from clinical studies could promote the analysis of microarray and gene expression. The analysis could be helpful for identifying the different pathways, molecular mechanisms, biological biomarkers and appropriate treatment. The investigations can be easily done at a low cost with less time using bioinformatics analysis.

**Figure 1:**
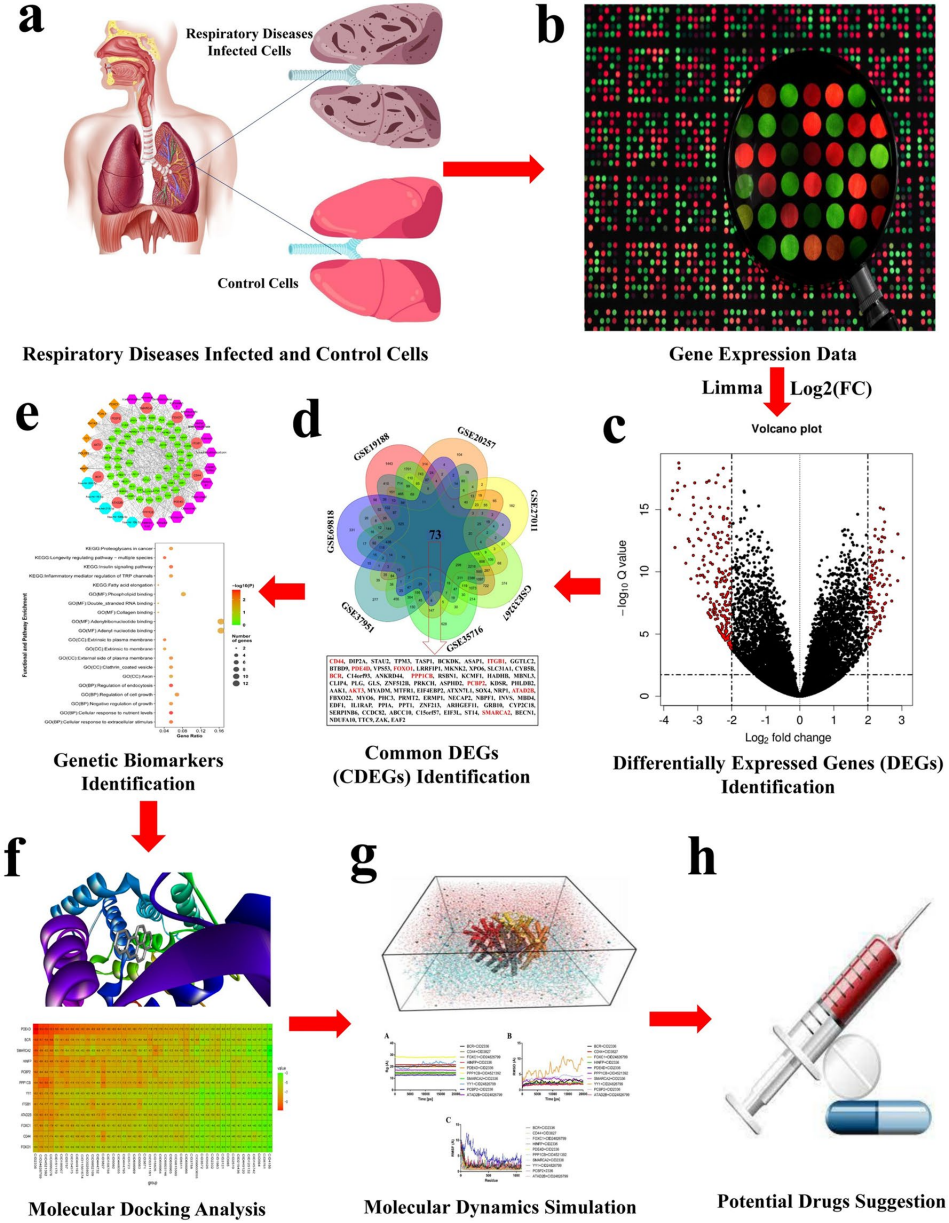
The schematic flowchart of the present study. (**a,b**) We first collected respiratory diseases (lung cancer, COPD, asthma, SARS-COV, pneumonia, influenza and emphysema) microarray gene expression datasets composed of control and infected samples and preprocessed them for analysis. (**c,d**) The collected datasets of respiratory diseases were analyzed to identify differentially expressed genes (DEGs) individually2, and then the common DEGs (CDEGs) among all the respiratory diseases. (**e**) Genetic biomarkers, e.g., Drug targets (Hub DEGs, TFs), miRNAs, drug agents (drugs, chemicals), gene ontology and pathways of respiratory diseases were identified using CDEGs through integrated bioinformatics analysis. (**f–h**) After identifying drug targets and agents, we conducted molecular docking and molecular dynamics simulation analysis to suggest potential drug complexes for respiratory diseases.

Recently, few molecular mechanisms or pathogenesis of the individual RD (asthma or COPD or lung cancer or other lung diseases) were identified. However, they have not studied the common molecular pathogenesis of the RD, which still needs to be identified^6–12^. In the present study, we would like to investigate the common molecular mechanisms of the RD by using microarray data through an integrated bioinformatics approach. This study attempts to find differentially expressed genes, HubGs, significant regulatory checkpoints, regulatory biomarkers, drug targets and drug agents. In the present study, we used seven datasets from the Gene Expression Omnibus (GEO) database (GSE19188, GSE20257, GSE27011, GSE33267, GSE35716, GSE37951 and GSE69818) of RD in the bioinformatics analysis. First, differentially expressed genes (DEGs) for the seven datasets were identified. Next, common DEGs (CDEGs) for the seven datasets were discovered. The most important task is to identify HubGs from frequent CDEGs using protein–protein interaction (PPI) network analysis. Also, the CDEGs are largely responsible for identifying regulatory checkpoints (gene ontology and pathway), pharmacological targets, drug agents (i.e., drugs and chemicals) and regulatory biomarkers (i.e., transcription factors and microRNAs). Finally, we screened potential drug targets (i.e. TFs and HubGs), drug agents and their compounds, which may be useful in fighting against all RD. The workflow of the present research is illustrated in Fig. 1.

## Materials and methods

### Data sources and identification of DEGs

To explore RD-related biological or molecular information, the human gene expression profile was considered. Gene expression profile datasets GSE19188^13^, GSE20257^14^, GSE27011^15^, GSE33267^16^, GSE35716^17^, GSE37951, and GSE69818^18^ were taken from the GEO database (http://www.ncbi.nlm.nih.gov/geo/)^19^ for lung cancer, COPD, asthma, SARS-COV, pneumonia, influenza, emphysema, respectively. Next, the datasets were analyzed to identify DEGs between RD-infected cases and control samples. The above datasets were analyzed through GEO2R^20^ web tool, where the expression matrix was log2-transformed, and *limma* R packages to identify DEGs with P-value < 0.05 and $|{log}_{2}{ FC}_{i}|>0$. Next, the common DEGs (CDEGs) were identified among the DEGs of each dataset, which were further visualized using the Venn diagram through the *“venn”* package in R programming language.

### Network-based molecular interaction analysis

To find out regulatory components and HubGs from the CDEGs, we investigated the network-based molecular interaction analysis, including CDEGs with transcription factors (TFs), micro-RNA (miRNA), drugs, chemical components and protein–protein interaction (PPI). The specific detailed analysis of these molecular interactions is discussed in the following sub-sections.

#### Protein–protein interactions (PPI) analysis of CDEGs

To analyze protein interactions, the PPI network was created. To create the PPI network, the CDEGs are inserted in the STRINGdb web portal (https://string-db.org/)^21^, with a confidence score of 0.9. To locate the HubGs in the PPI networks, we employed a topological degree of measurement (> 11).

#### Interaction analysis of CDEGs with their regulatory factors

Regulatory biomarkers (i.e., TFs and miRNAs) are control genes at the post-transcriptional and transcriptional level in plenty of cellular functions and biological activities^22,23^. The regulatory biomarker TFs-CDEGs and miRNAs-CDEGs network information were collected using the NetworkAnalysis tool^24^ and visualized by Cytoscape^25^. We created the TFs-CDEGs interactions network based on the JASPAR database^26^ and miRNAs-CDEGs interactions from TarBase^27^ and miRTarBase^28^ databases. From the networks, we find out the important TFs and miRNAs based on their highest topological degree. Next, top TFs and miRNAs were chosen as the regulators of the discovered CDEGs. Moreover, for the identified important miRNAs, we conducted an over-representation analysis (ORA) with a set of diseases based on miRNA-Disease association information by using miEAA web server [version 2.1]^29^.

#### Interaction analysis of CDEGs with their toxicogenomics and pharmacogenomics factors

To meet expanding demand in pharmacogenomics and toxicogenomics, we also analyzed CDEGs-chemical and CDEGs-drugs interaction networks by using the NetworkAnalysis tool^24^. The CDEGs-chemical interaction network was performed from the Comparative Toxicogenomics Database (CTD)^30^ and the CDEGs-drugs interaction network was performed from DrugBank^31^ database. The significant chemicals were selected according to a topological degree > 25 and cDEGs-related drugs were found in the network.

#### Functions and pathway enrichment analysi*s* of CDEGs

The principal reason for identifying functional and pathway enrichment terms is to understand the molecular action, cellular role and place in a cell. This study employed to identify significant functions and pathway enrichment analysis of CDEGs utilizing the Gene Ontology (GO)^32^ and Kyoto Encyclopedia of Genes and Genomes (KEGG) databases^33^ through NetworkAnalyst. The statistical significance of the functional enrichment study was evaluated using a Fisher exact test with a cut-off p-value < 0.05.

### Molecular docking simulation of proteins with aspirant drugs

We used molecular docking simulation between the drug target and drug agents in order to suggest effective medicines for the RD. We used the 63 drug agents or ligands determined by CDEG-chemical and CDEG-Drug network and our hypothesized hub-proteins and TFs proteins were used as the drug target. From the PubChem^34^ database, the 3D appropriate structures of 58 FDA-approved ligands were obtained, as seen in Table S1. AutoDock Vina was utilized for docking studies and virtual screening of pharmacological agents to calculate the binding affinities (kcal/mol) of docked complexes^35^. The exhaustiveness value was set to −8.0 (kcal/mol). The structure was generated, and the 2D protein–ligand interactions were visualized using the Discovery Studio Visualizer interface^36^.

### Molecular dynamic simulation of the docked complexes

To validate the molecular docking study, we a performed molecular dynamics (MD) simulation of the docked complexes, which was implemented in the YASARA dynamics commercial package^37^. Initially, the complexes were loaded into the simulation system, and the AMBER14 force field was used to minimize system energy^38^. In addition, water molecules and 0.9% NaCl were added at 310 K for system neutralization. To calculate the long-range electrostatic interaction, the particle mesh Ewald method (PME) was applied^39^. A cubic simulation cell was set up by 20 Å, and the system temperature was controlled by the Berendsen thermostat. The energy minimization of the system was carried out through the simulated annealing method^40^. The normal simulation time step (1.25 fs) was set, and trajectories were collected for every 100 ps. Finally, molecular dynamic simulation of the complexes was run for 100 ns and the radius of gyration (Rg), root mean square deviation (RMSD) and root mean square fluctuation (RMSF) were calculated to understand the conformational stability and variation of the complex. It has become common practice to use the "Molecular Mechanics Poisson-Boltzmann Surface Area" (MM-PBSA) approach to determine the binding free energy of the protein–ligand complex^41^. Using the YASARA structure, the MM-PBSA has been used to calculate the binding free energy of the protein–ligand complex. The few snapshots (~ 100 ns) of the dynamic simulation trajectory were used to determine the MM-PBSA of the protein–ligand complex structure. The protein–ligand complex's binding free energy can be computed as follows:

$${\Delta E}_{\mathrm{MM}-\mathrm{PBSA}}={E}_{\mathrm{complex}}-{(E}_{\mathrm{protein}}+{E}_{\mathrm{ligand}})$$

where, ${E}_{\mathrm{complex}}$ is the total MM-PBSA energy of protein–ligand complex, ${E}_{\mathrm{protein}}$ and ${E}_{\mathrm{ligand}}$ are the total solution free energies of the isolated protein and ligand, respectively.

## Results

### Identifications of DEGs in RD

**Figure 2:**
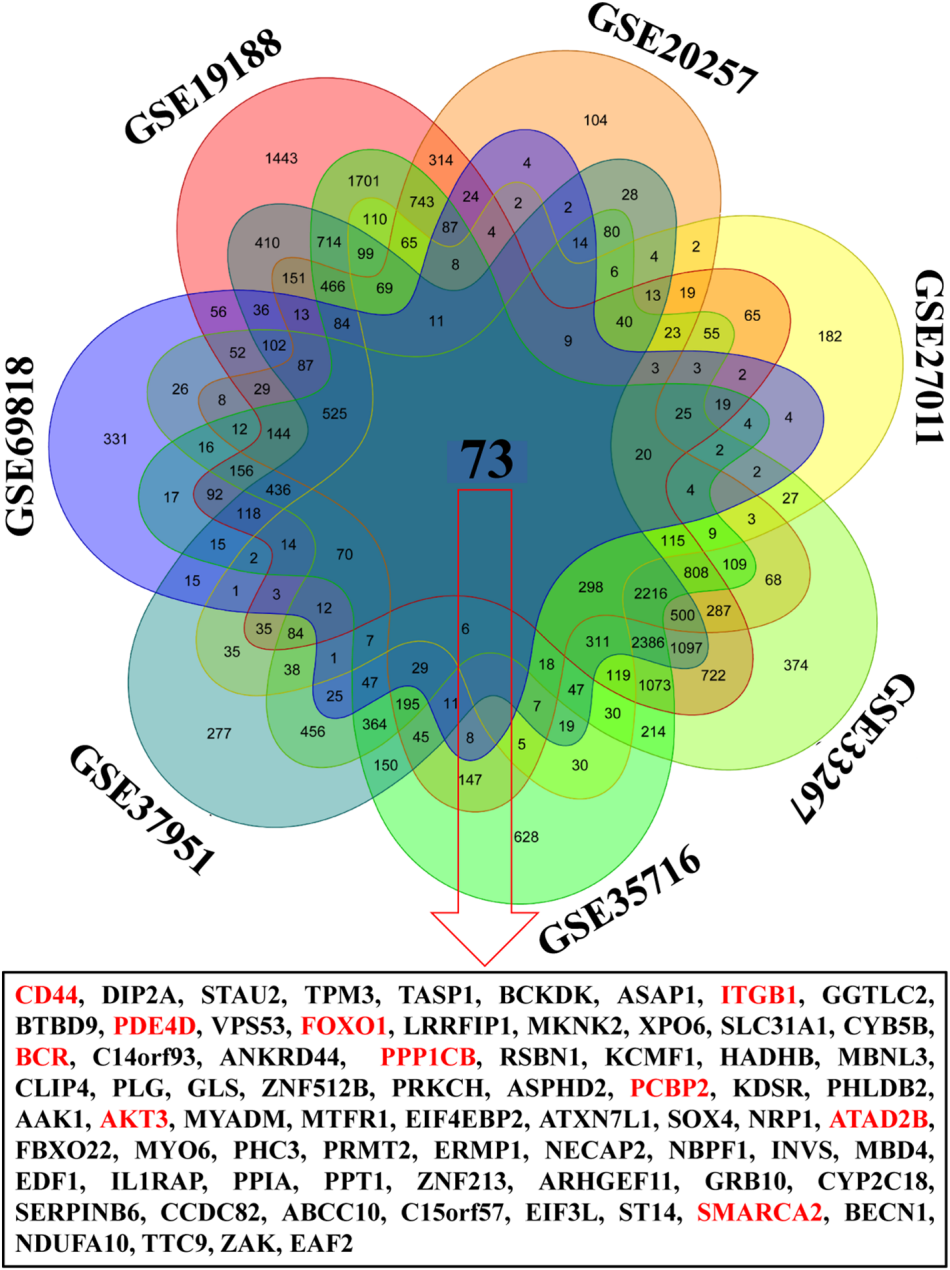
The CDEGs representation through a Venn diagram. List of significant CDEGs (73) for RD where red color is denoted HubGs.

The gene expression dataset of seven RD datasets, i.e., GSE19188, GSE20257, GSE27011, GSE33267, GSE35716, GSE37951, and GSE69818 were used for the identification of DEGs, and a total of 18868, 8177, 2292, 14115, 15372, 12425 and 2931 DEGs were found, respectively. Collected DEGs for RD were identified by using R programming language and 73 common DEGs (CDEGs) were found. In Fig. 2, a Venn diagram is used to visualize the shared DEGs across these datasets. According to the Venn diagram results, there are 23182 different DEGs in total, with 0.31% of them being common.

### Protein–protein interactions (PPI) analysis of CDEGs

**Figure 3:**
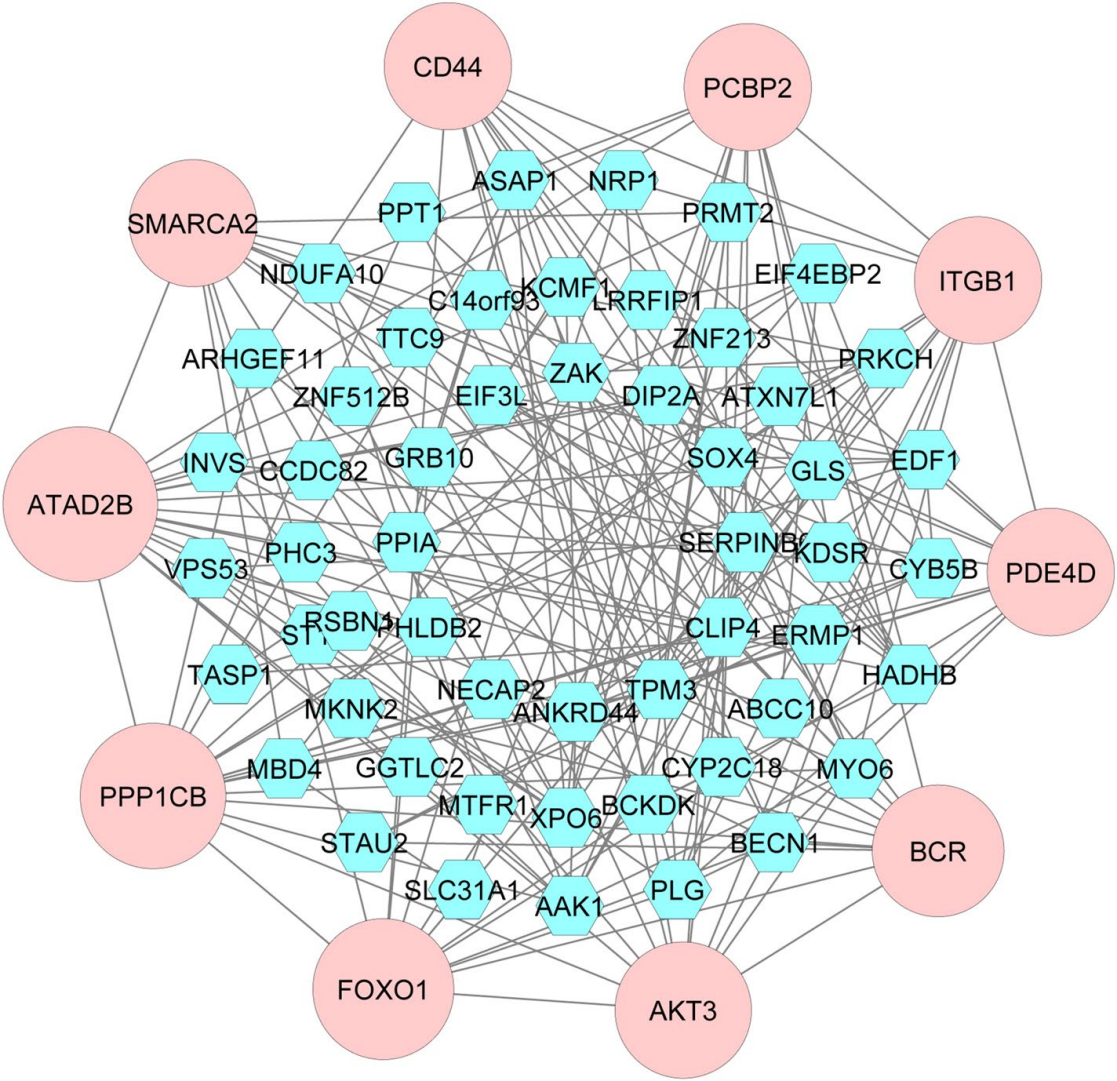
PPI network for identified CDEGs that are shared by the RDs. The larger nodes highlighted with pink color indicate the HubGs and the edges specify the interconnection in the middle of the two genes.

The 73 CDEGs were used to build a PPI network in order to identify the hub genes (HubGs). We input CDEGs in the STRING database to collect interconnected proteins. According to the topological measure degree (> 11), the list of HubGs was chosen, as displayed in Fig. 3. The identified 10 HubGs are *ATAD2B, PPP1CB, FOXO1, AKT3, BCR, PDE4D, ITGB1, PCBP2, CD44* and *SMARCA2*.

### Functional and pathway enrichment analysis of CDEGs

**Figure 4:**
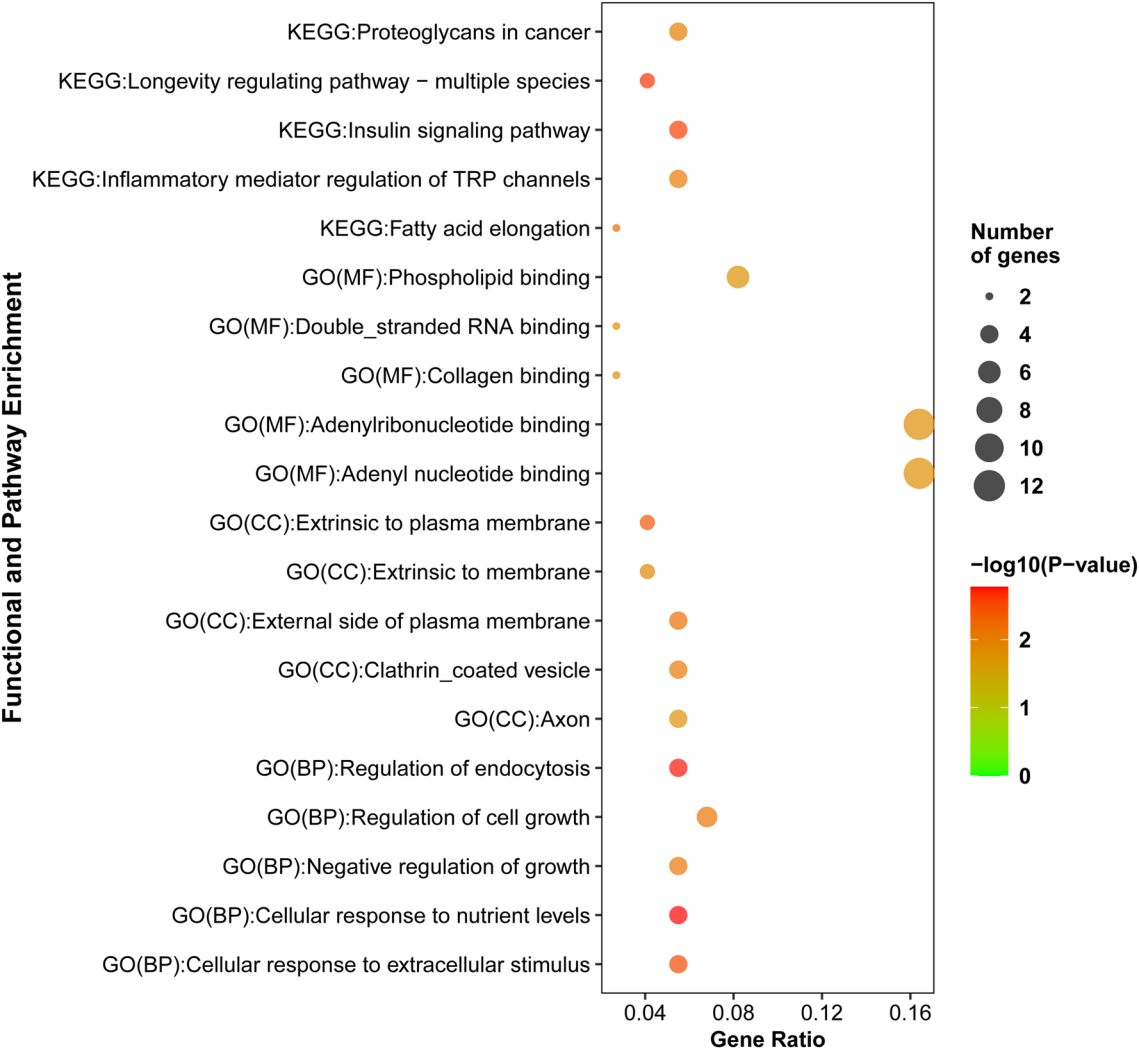
The top five significantly enriched GO terms and KEGG pathways that are involved in the pathogenesis processes of RD. Here, the point size and colour depend on involved gene numbers and the significance of the enrichment analysis (p-value).

We carried out KEGG pathway and GO categories enrichment analyses to examine the biological roles of the CDEGs in more detail. Here, the GO-term enrichment analysis was performed by NetworkAnalyst. Here, we showed the top 5 significantly enriched terms in Biological Process (BP), Molecular Functions (MF), and Cellular Component (CC), which interacted with CDEGs (Fig. 4 and Table S2).

We identified the top five BP: C*ellular response to nutrient levels, Regulation of endocytosis, Cellular response to extracellular stimulus, Regulation of cell growth* and *Negative regulation of growth*. We also identified the MF, including *Collagen binding, Adenyl ribonucleotide binding, Double-stranded RNA binding, Phospholipid binding,* and *Adenyl nucleotide binding*. We then identified the top five CC: *Extrinsic to the plasma membrane, External side of the plasma membrane, Clathrin coated vesicle* and *Extrinsic to membrane and Axon*.

In the KEGG pathway analysis, we exposed significantly top 5 enriched pathways in (Fig. 4 and Table S2). The top 5 enriched pathways include *Longevity regulating pathway-multiple species, Insulin signalling pathway, Fatty acid elongation, Inflammatory mediator regulation of TRP channels* and *Proteoglycans in cancer*.

### Different interaction and network analysis of CDEGs

#### Interaction analysis of CDEGs with their regulatory factors

**Figure 5:**
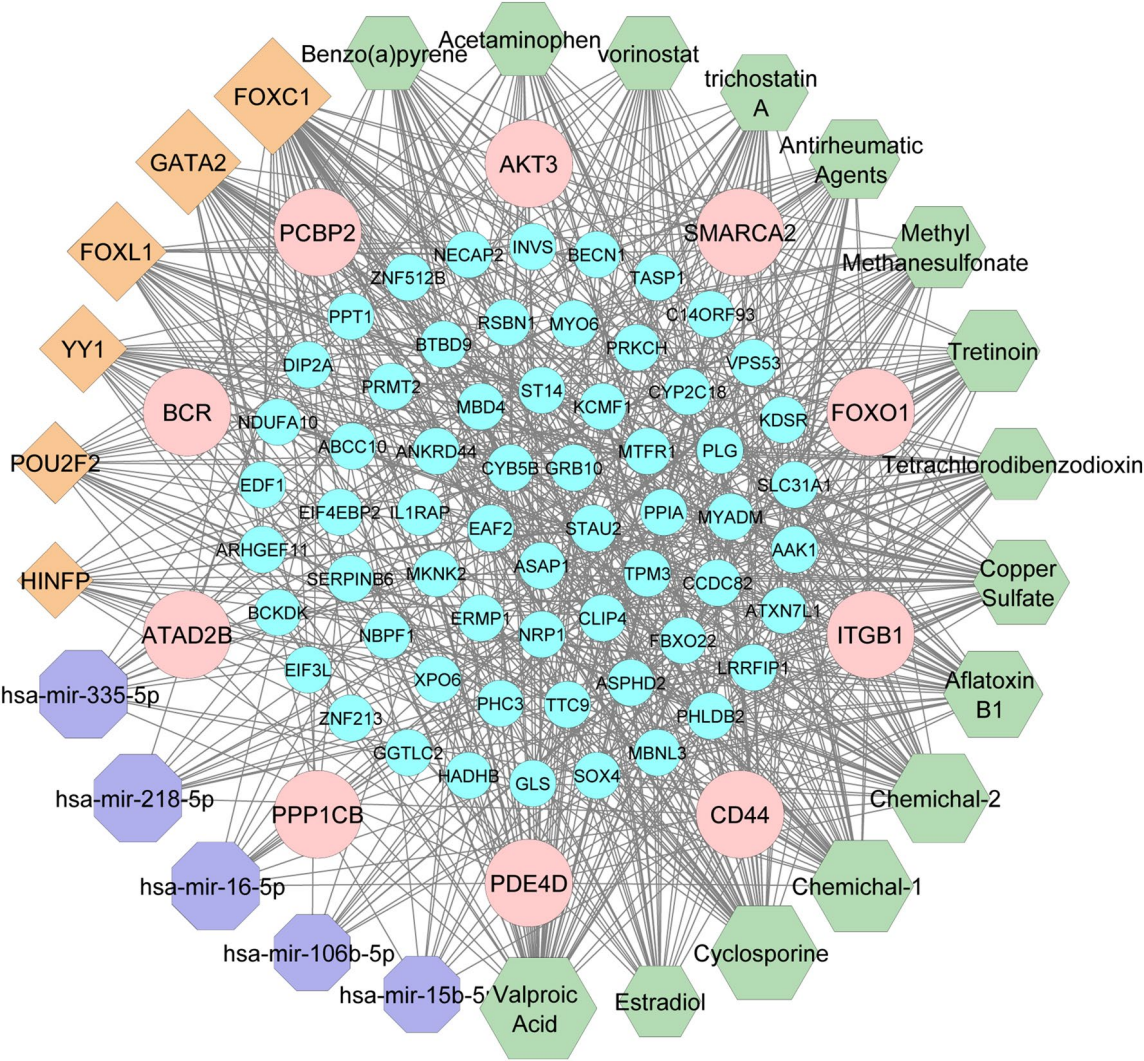
Interaction network for regulatory elements with CDEGs. Here miRNAs are indicated with octagon shape, chemicals with hexagon shape, TFs with square shape, HubGs with pink colour and circle shape, and DEGs are indicated with sky-blue colour and circle shape. Here, the two chemical agents are *4-(5-benzo(1, 3)dioxol-5-yl-4-pyridin-2-yl-1H-imidazol-2-yl)benzamide*, and *(6-(4-(2-piperidin-1-ylethoxy)phenyl))-3-pyridin-4-ylpyrazolo(1, 5-a)pyrimidine.*

TFs and miRNAs control the transcriptional and post-transcriptional phases of gene regulation. To identify key regulators (TFs and miRNAs), we have established networks of CDEGs-TFs and CDEGs-miRNAs. This list of related TFs and miRNAs is summarized in Table S3 and Table S4, respectively. The analyses of the CDEGs-TFs network found seven TFs according to a number of associated CDEGs (≥ 18), namely *FOXC1, GATA2, FOXL1, YY1, POU2F2*, and *HINFP* as shown in Fig. 5. The CDEGs-miRNAs network found the top 5 miRNAs according to a number of associated CDEGs ($>$ 10), namely *hsa-mir-218-5p, hsa-mir-335-5p, hsa-mir-16-5p, hsa-mir-106b-5p,* and *hsa-mir-15b-5p* in Fig. 5. Based on miRNA-Disease association information with FDR-adjusted p-values < 0.05 by using miEAA web server, a set of respiratory diseases were found highly enriched, e.g., Pulmonary fibrosis (FDR-adjusted P-value: $3.54 \times {10}^{-4}$), Lung small cell carcinoma (FDR-adjusted P-value: $3.42 \times {10}^{-4}$), Chronic obstructive pulmonary disease (FDR-adjusted P-value: $2.14 \times {10}^{-3}$), Lung squamous cell carcinoma (FDR-adjusted P-value: $2.15 \times {10}^{-3}$), Lung cancer (FDR-adjusted P-value: $2.94 \times {10}^{-3}$), Pulmonary tuberculosis ($\mathrm{FDR}-\mathrm{adjusted P}-\mathrm{value}: 8.21 \times {10}^{-3}$), Asthma (FDR-adjusted P-value: $1.4 \times {10}^{-2}$), Pulmonary embolism (FDR-adjusted P-value: $3.42 \times {10}^{-4}$), and Hypoxia (FDR-adjusted P-value: $3.42 \times {10}^{-4}$), which indicates the significant association between identified miRNAs and their roles in respiratory diseases.

#### Interaction analysis of HubGs with their toxicogenomic and pharmacogenomic factors

The toxicogenomic and pharmacogenomic phases are controlled by chemicals and drugs, respectively. The CDEGs-chemicals and CDEGs-drugs interactions were collected using NetworkAnalyst and visualized in Fig. 5**.** The analyses of the CDEGs-chemicals network found 15 chemicals according to a number of associated CDEGs ($>$ 25), which is arranged in Table S5. Also, the analyses of the CDEGs-drugs network found 48 drugs which are significantly interacted with CDEGs of RD. The list of connected drugs is summarized in Table S6.

### Drug repurposing by molecular docking

**Figure 6:**
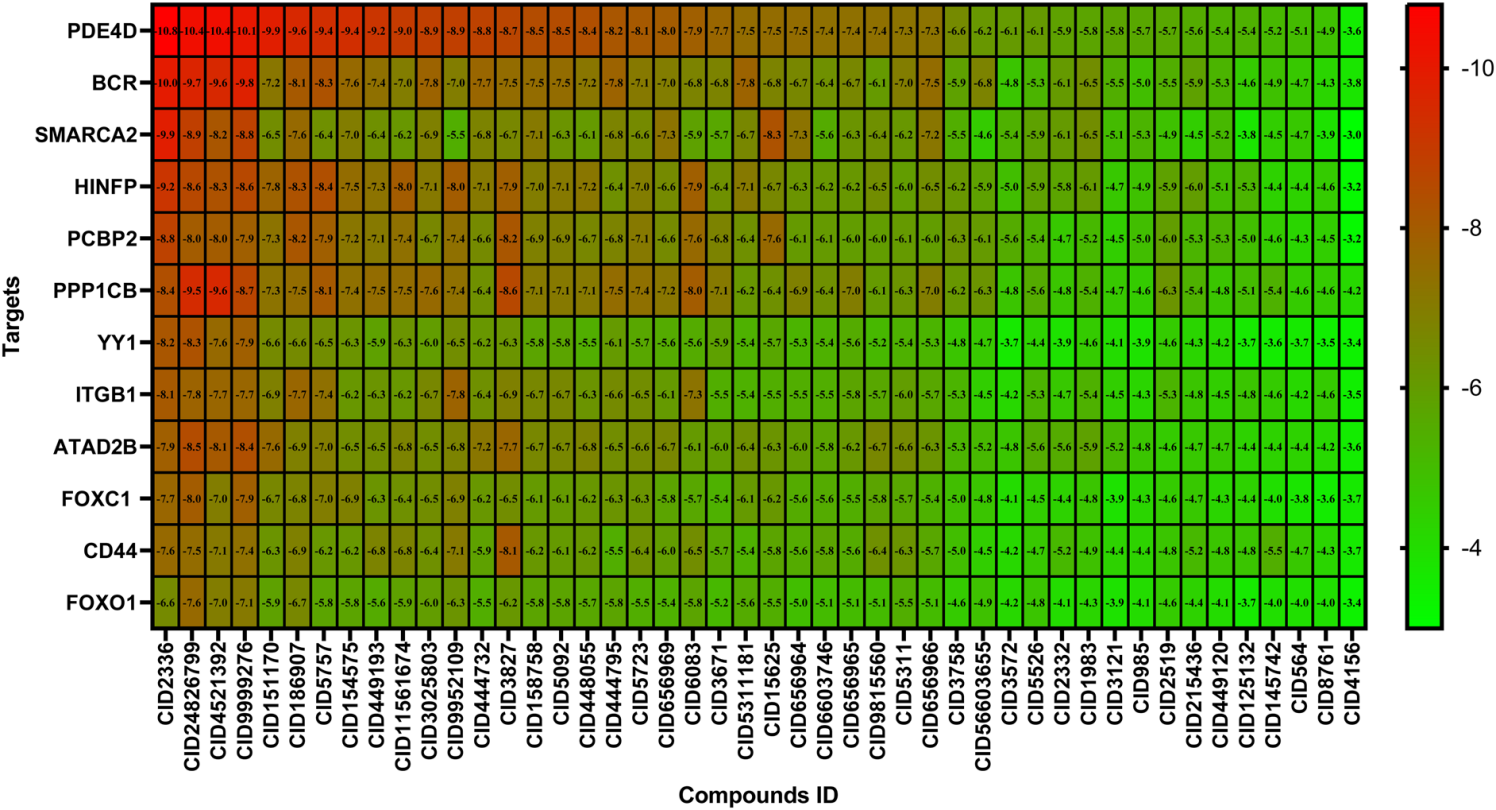
Molecular docking simulation results by Autodock Vina. Red colours indicated the strong binding affinities between target proteins and drug agents, and green colors indicated their weak bindings. Image of binding affinity scores based on the proposed drug agents in the X-axis and 12 target proteins (proposed) in the Y-axis.

We took the proposed 10 HubGs and 6 key TF proteins as 16 drug target receptors as well as the recommended 15 chemicals and 48 drugs as ligands. The 3D structure of target proteins (*ATAD2B, PPP1CB, FOXO1, AKT3, BCR, PDE4D, ITGB1, PCBP2, CD44, SMARCA2, FOXC1, YY1,* and *HINFP*) were retrieved from Protein Data Bank (PDB)^42^ with the PBD codes 3LXJ, 1s70, 3CO6, 3CQW, 3OXZ, 3G4L, 4WK0, 2PQU, 1uuh, 5DKC, 6akP, 1UBD, and 3CFS, respectively. The 3D structures of the proposed 63 drug agents were taken from the PubChem database^34^ listed in Table S1. In the molecular docking simulations, we observed and selected only the highest binding affinity drug agent with every proposed protein, which produces at least negatively larger than -8.0 kcal/mol. Therefore, we considered the top-ranked 11 docked that lead to complexes, namely, *BCR* + CID2336, *CD44* + CID3827, *FOXC1* + CID24826799, *ITGB1* + CID2336, *HINFP* + CID2336, *PDE4D* + CID2336, *PPP1CB* + CID4521392, *SMARCA2* + CID2336, *PCBP2* + CID2336, *ATAD2B* + CID24826799, and *YY1* + CID24826799 as the most plausible candidate drugs displayed in Table S7 and Fig. 6. Table S7 shows the binding pose of a protein–ligand complex of the proposed 11 potential complex, 3D structures of drug target and neighboring residues of the proteins with prospective drug interaction.

### Protein–ligand complex stability and conformational flexibility by MD simulation

The MD simulation can be used in computer-aided drug discovery. The simulation was run to validate the molecular docking study. The Rg, RMSD, RMSF, and MM-PBSA of the protein–ligand complex were used to examine the conformational stability and variation of the complex. For this simulation, we considered the proposed 11-docked lead complex.

#### Radius of gyration (Rg)

**Figure 7:**
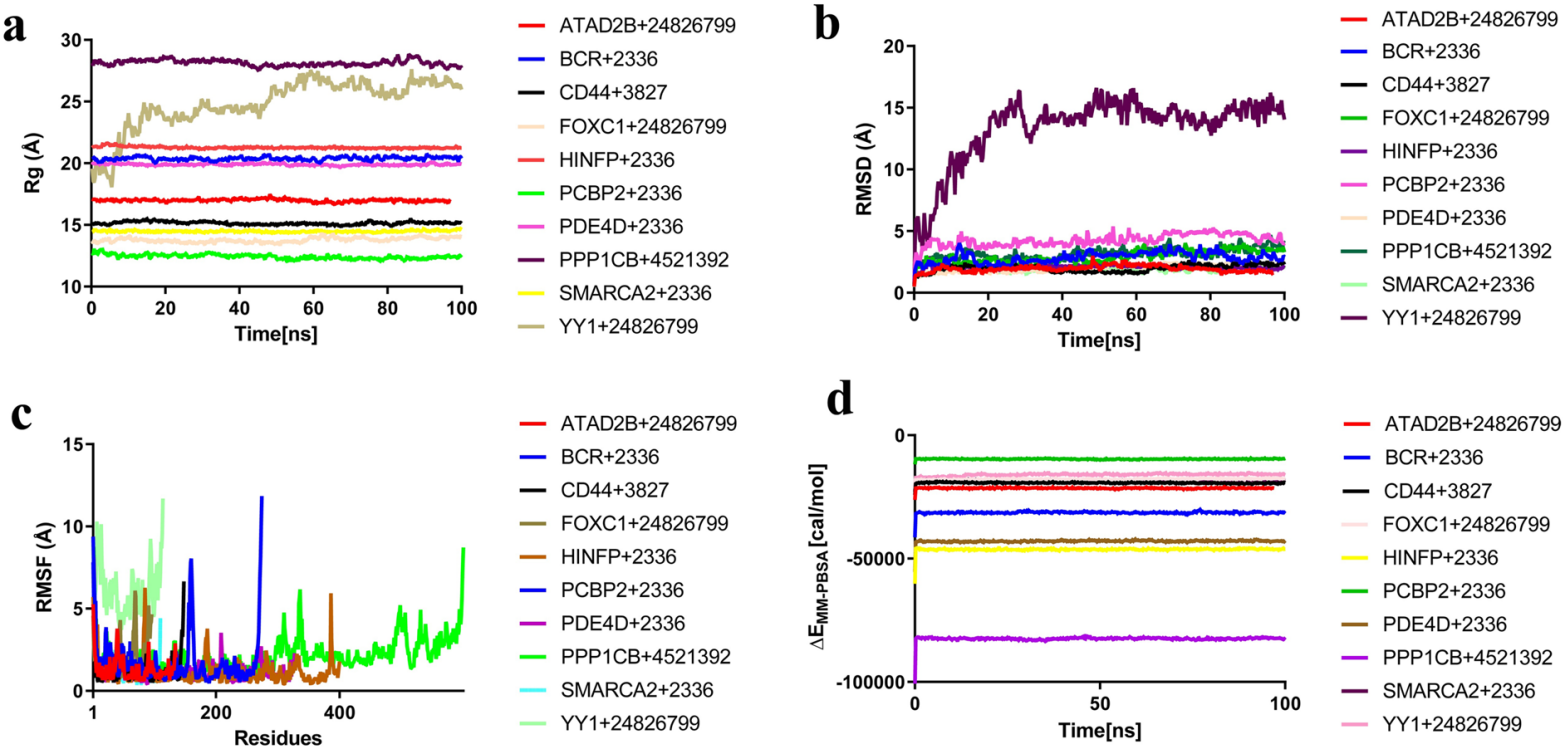
The results from molecular docking performance and MD simulation showing the (**a**) radius of gyration (Rg), (**b**) RMSD and (**c**) RMSF and (**d**) MM-PBSA analysis of the selected protein–ligand docked complex according.

The Rg of the protein–ligand complex has been calculated to predict a macromolecule's structural behavior. It also shows variations of complicated compactness of the molecules. In Fig. 7a, the stability of the chosen complexes was also investigated in terms of Rg throughout the 100 ns simulation. The difference (Standard deviation) of Rg was found to be 0.843 (0.144), 0.671(0.125), 1.031 (0.191), 0.553 (0.0852), 0.429 (0.0739), 1.29 (0.246), 0.478 (0.08), 1.057 (0.182), and 0.85 (0.118) Å for the complexes *BCR* + CID2336, *CD44* + CID3827, *FOXC1* + CID24826799, *HINFP* + CID2336, *PDE4D* + CID2336, *PPP1CB* + CID4521392, *SMARCA2* + CID2336, *PCBP2* + CID2336, and *ATAD2B* + CID24826799, respectively, which suggests that the above-mentioned complexes are steady. However, the difference of Rg with the value of 9.572 Å and the standard deviation of 1.995 Å for the *YY1* + CID2482679, suggests that the complex is unstable.

#### Root mean square deviation (RMSD) Analysis

The RMSD of Cα atoms have been computed for the proposed protein–ligand complexes to measure the protein structure stability. The average RMSD value of *ATAD2B* + CID24826799, *CD44* + CID3827, *HINFP* + CID2336, *PDE4D* + CID2336 and *SMARCA2* + CID2336 are 1.945 Å, 1.997 Å, 2.067 Å, 2.009 Å and 1.896 Å, respectively. The five complexes are stable among the selected complexes, where they also show a good RMSD within a range of 0.477–2.997 Å and low RMSD difference of the complex system (Fig. 7b). However, other selected complexes, i.e., *BCR* + 2336, *FOXC1* + 24826799, *PCBP2* + CID2336, PPP1CB + CID4521392 and *YY1* + CID24826799 became unstable among other complexes, where it shows unacceptable RMSD values (greater than 3.0 Å) and also shows very high deviation.

#### Root mean square fluctuation (RMSF) analysis

The fluctuation and stability level of amino acid (AA) residues in a complex system are determined by the high RMSF value of the amino acid residues. Among the complexes, CD44 + CID3827 all the areas of the AA residue in the protein structure do not show fluctuation during the simulation. The *FOXC1* + CID24826799, *ATAD2B* + CID24826799, *SMARCA2* + CID2336, *PPP1CB* + CID4521392, *PDE4D* + CID2336, *BCR* + CID2336, *HINFP* + CID2336 and *PCBP2* + CID2336 complexes displaythe maximum area of the AA residue in their respective protein structures, which did not demonstrate fluctuation during the simulation (Fig. 7c). But on the other hand, in the case of the *YY1* + CID2482679 complex, all the area of the AA residue in the protein structure exhibitsfluctuation during the simulation.

#### Molecular mechanics Poisson–Boltzmann surface area (MM-PBSA) analysis

The binding free energy of the protein–ligand complex has been calculated using the MM-PBSA. The higher net negative binding free energy values are −26.243 kcal/mol, −41.358 kcal/mol, −24.236 kcal/mol, −21.714 kcal/mol, −60.238 kcal/mol, −11.795 kcal/mol, −55.428 kcal/mol, −102.544 kcal/mol, −22.914 kcal/mol and −20.946 kcal/mol for the selected complex *ATAD2B* + CID24826799, *BCR* + CID2336, *CD44* + CID3827, *FOXC1* + CID24826799, *HINFP* + CID2336, *PCBP2* + CID2336, *PDE4D* + CID2336, *PPP1CB* + CID4521392, *SMARCA2* + CID2336 and *YY1* + CID24826799, respectively (Fig. 7d). As a result, it is reasonable to predict that the complexes will be able to maintain a sustained relationship.

## Discussion

Respiratory Diseases (RDs) affect the airways and other structures of the lungs. Some of the most common are asthma, COPD, lung cancer and other lung diseases. Currently, a number of treatments are available that can assist in managing symptoms and consequently ensuring better daily living. However, RDs are currently not fully curable (https://www.who.int). The objective of our study is to detect common differential genes and potential HubGs, emphasizing their actions, signaling pathways, regulatory biomarkers for diagnosing RD, and potential candidate drugs for the treatment of RD. The study aims to reduce the toll of morbidity, disability and premature mortality by RD. In this study, we used the bioinformatics approach to examine the molecular mechanism of RD from gene expression data. We first identified common differentially expressed genes. In addition, we also focused on identifying responsible TFs, miRNAs, chemicals and HubGs by generating different interactions with the CDEGs, which play key roles in the regulation of CDEGs and drug development for RD. Later, we structured functional and pathway enrichment analysis with the CDEGs to determine the biological roles of CDEGs in those RD. Finally, we repositioned the drug candidate of RD through molecular docking and molecular dynamics simulation.

The identified HubGs may be responsible for infecting RDs in the human body. Previous studies have shown that PDE4D antagonists may be a possible treatment for chronic airway illnesses^43,44^. Gene array and bioinformatics analyses implied that *ITGB1* protein expression levels were higher in lung cancer patients^45^. Through network analysis, the identified *ITGB1* protein was considered a drug target for preventing and treating COVID-19 patients in the past^46^. The *CD44* is a potential biomarker of a number of diseases, including lung disease and pneumonia^47,48^. *SMARCA2* is identified as a tumor suppressor gene whose expression was regulated in lung cancers and influenza^49,50^. The *BCR* and *AKT* can be novel therapeutic targets for RD suggested by the study conducted by some authors^51,52^. Asthma, COPD and pneumonia are associated with PCBP2 found in the previous study^53^. The *FOXO1* is implicated in human lung carcinogenesis and has a preventive effect in oxidative settings in COPD^54^. These functions make it a viable therapeutic target for the spread of human lung cancer.

In this study, we identified key TFs and miRNA which are very essential to identify the nature of RD. The FOXC1 is a model hypoxia-induced transcription factor that is essential in promoting lung cancer cells' growth, migration, invasion, angiogenesis, and transformation from epithelial to mesenchymal state. The FOXC1 TF may be an active therapeutic target for lung cancer^55^. The regulatory network analysis from the existing studies showed that *FOXC1, GATA2, YY1,* and *FOXL1* are significant TFs for COVID-19^56^. In addition, the *GATA2* reduction has a direct relationship to diffuse parenchymal lung disease. The expression of the transcription factor *GATA2* is differentiable in lung disease^57^. A previous study suggested that *FOXL1* and *YY1* regulate multiple functional aspects of lung fibroblasts as a key transcription factor and are involved in idiopathic pulmonary fibrosis pathogenesis^58,59^. The *YY1* modulates Lung Cancer Progression and other lung diseases^60^. *POU2F2* could function as a potential therapeutic target for lung cancer because it was significantly expressed in lung cancer cells^61^.

It is significant that miR-218-5p expression and airway blockage have a substantial correlation. In the aetiology of RD, miR-218-5p may have significant effects^62^. MiR-335-5p expression levels were linked to respiratory illnesses in a previous study^63^. MiR-16-5p and miR-106b-5p targets were related to response to influenza^64^. The miR-16-5p may be associated with lung injury and play a role as a prognostic biomarker^65^. According to reports, miR-106b-5p is essential for the physiological operation of lung cancer, which may result in a novel treatment for RD^66^. Previous studies have indicated that miR-15b-5p and miR-16-5p may be potential markers in the diagnosis of lung cancer at an early stage^67^. The hsa-miR-15b-5p was a potential biomarker of COPD and influenza^68,69^. The discussion and miRNA-Disease association analysis indicates that there is a strong connection between the functions played by discovered miRNAs in respiratory disorders.

After that, we performed GO categories and KEGG pathway enrichment analysis to investigate key functions and pathways. Cellular response to nutrient levels plays a key role in nutritional immunity in the response of the lung to infection and chronic RD^70^. A vital cell process known as endocytosis may be involved with a novel treatment strategy to manage respiratory infections^71^. Extracellular vesicles are involved in viral infection, pathogeneses, diagnoses and treatment of chronic lung diseases of lung injury and inflammation^72^. Regulation of cell growth involved in acute lung injury, lung cancer progression and inflammation^73^. Collagen is the main ECM component and hence plays a critical role in lung development, pathogenesis and progression of chronic lung diseases^74^. Adenylribonucleotide binding may play a role as a key pathway of acute lung disease^75^. Double-stranded RNA is required for innate immune and antiviral response in respiratory epithelial-derived cells and plays an important role in lung diseases^76^. The phospholipid imbalance causes lung diseases and shows a defense mechanism against pulmonary infections^77^. Phospholipid is also involved in immune protection against respiratory viral infection^78^. Adenyl nucleotide binding was one of the pathways for investigating mechanisms in the treatment of lung cancer^79^. The plasma membrane is related to pathogenic mechanisms of cell wounding in lung diseases^80^. The clathrin-coated vesicle cycle pathway was significantly associated with RD^81^. Axon-like protrusions promote lung cancer migration and metastasis, and they might influence the behavior of lung diseases^82^. Insulin signaling is required for lung development and inflammatory lung diseases^83^. In the same way, fatty acids lead to the development of chronic lung diseases^84^. It can be used in the prevention and treatment of diseases. Inflammatory mediator regulation of TRP channels plays an important role in airway diseases/chronic lung diseases^85^. Proteoglycans in cancer may serve as a biomarker for tumor progression and patient survival since it promotes lung cancer cell migration^86^. Proteoglycans are a key regulator of pulmonary inflammation and the innate immune response to lung infection^87^.

We picked the ten key proteins and their regulatory 6 TFs proteins as the drug target receptors as well as conducted their docking analysis with 63 drug agents. Then we selected the top-ranked five protein-drug complexes as the most probable repurposable candidate drugs complexes for RD based on their strong binding affinity scores (kCal/mol) and molecular dynamics simulation. Among the identified candidate drugs, Benzo[a]pyrene might be encouraged RD^88^. However, in the case of infected lungs, where the genes are differentially expressed, the Benzo[a]pyrene may be used as a therapeutic agent for the treatment of lung diseases, as found in our docked and molecular dynamics study. The potential role of benzo[a]pyrene leads to inducing COPD, and pulmonary inflammation^89,90^. Benzo[a]pyrene plays a key role in inducing lung cancer^91^. According to molecular docking results we can say that the benzo[a]pyrene can bind well to PDE4D, SMARCA2 and HINFP in the wet lab since the binding affinity scores were −10.8 kcal/mol, −9.9 kcal/mol and −9.2 kcal/mol, respectively which is a better result. The three complexes can show high compactness since the complexes showed low and stable Rg values. The RMSD (1.896–2.067 Å) and RMSF scores of the protein–ligand complex indicate that the complexes could show better stability and would not show fluctuation. According to a prior study, ketotifen lessens obstructions of the conducting airways and may possibly have a direct impact on the small airways^92^. After a therapeutic trial, it was found that ketotifen shows a good impact on the asthmatic patient^93^. It has an antiviral activity that may play a potential role in the treatment of SARS-CoV-2 infection in humans^94^. Ketotifen contributes to reducing end-organ damage and mortality of mice infected by Influenza^95^. We may conclude from molecular docking studies that ketotifen can bind to CD44 in a satisfactory manner in a wet lab setting because the binding affinity score was −8.1 kcal/mol. It may show high compactness for low and stable Rg values of protein–ligand complexes. We may also draw the conclusion that the complex would not exhibit fluctuation because, in the simulation, none of the AA residue-containing regions in the protein structure exhibit fluctuation. The average RMSD value (1.997 Å) of the complex are specified that the complexes could show better stability. In previous research, the author’s shortlisted two FDA-approved drugs, where ponatinib is one of them to treat COVID-19 therapeutics^96^. According to the published study, ponatinib may serve as a new immunomodulator in the treatment of influenza, and it may be also effective for the treatment of lung cancer patients^97,98^. The binding affinity score was −8.5 kcal/mol which recommends that ponatinib may bind to ATAD2B well in a wet lab setting based on molecular docking studies. The low and stable Rg values of the ATAD2B-ponatinib complex suggest that the complex may exhibit high compactness. The protein–ligand complex's RMSD (1.945 Å) and RMSF scores suggest that the complex may be more stable and would not fluctuate. In order to effectively prevent RD, new technologies are being developed due to the difficulty of diagnosing and treating lung diseases. In light of this, the overall goal is defined as an integrative bioinformatic approach that includes multimodal diagnostics and disease-specific biomarker patterns. However, our identified biological information may shed light on the cause and progression of RD, as well as any new prospective therapeutic strategies.

## Conclusion

Interaction network for regulatory elements with CDEGs exposed the *hsa-miR-218-5p, hsa-miR-335-5p, hsa-miR-16-5p, hsa-miR-15b-5p* and *hsa-miR-106b-5p* which are associated with lung injury and may play a role as a prognostic biomarker. Interconnection of significant key functions and pathway enrichment with CDEGs revealed the five biological processes (*Cellular response to nutrient levels, Regulation of endocytosis, Cellular response to extracellular stimulus, Regulation of cell growth, Negative regulation of growth*), five molecular functions (*Collagen binding, Adenylribonucleotide binding, Double-stranded RNA binding, Phospholipid binding, Adenyl nucleotide binding*), five cellular components (*Extrinsic to the plasma membrane, External side of plasma membrane, Clathrin-coated vesicle, Extrinsic to membrane, Axon*) and five pathways (*Longevity regulating pathway—multiple species, Insulin signaling pathway, Fatty acid elongation, Inflammatory mediator regulation of TRP channels, Proteoglycans in cancer*). The functions and pathways may play an important role in airway diseases/chronic lung diseases. According to interaction network, molecular docking and molecular dynamics simulation identified the *PDE4D*-benzo[a]pyrene, *SMARCA2*-benzo[a]pyrene, *HINFP*-benzo[a]pyrene, *CD44*-ketotifen and *ATAD2B*-ponatinib as potential protein-drug complexes that will help to inhibit the RD. Therefore, the suggested molecular biomarkers and repurposing candidate drugs will be beneficial for the diagnosis and treatment of RD. The activity of drug complexes and biomarkers can be determined by further analysis using various lab-based trial techniques. The findings from this study will help to open up new avenues for cutting-edge treatments to reduce morbidity and future premature mortality from RD.

### Supplementary Information

Supplementary Tables.

## Data Availability

The Gene expression profile datasets GSE19188^13^, GSE20257^14^, GSE27011^15^, GSE33267^16^, GSE35716^17^, GSE37951, and GSE69818^18^ were taken from the National Center for Biotechnology Information (NCBI) Gene Expression Omnibus (GEO) open access database, where datasets are freely available (http://www.ncbi.nlm.nih.gov/geo/).
