# Supplementary material for: Identification of genetic biomarkers, drug targets and agents for respiratory diseases utilising integrated bioinformatics approaches
Source: Sci Rep. 2023 Nov 4;13:19072. doi: 10.1038/s41598-023-46455-8 (PMC10625598; doi:10.1038/s41598-023-46455-8)
Supplement: Supplementary file 1 — Supplementary Tables. [file 41598_2023_46455_MOESM1_ESM.pdf]

Table S1: FDA-approved ligands list

	Label	Pubchem ID
1	Adenosine monophosphate	CID 6083
2	Caffeine	CID 2519
3	Dyphylline	CID 3182
4	Ketotifen	CID 3827
5	Iloprost	CID 5311181
6	Roflumilast	CID 449193
7	Piclamilast	CID 154575
8	Rolipram	CID 5092
9	3,5-Dimethyl-1-(3-Nitrophenyl)-1h-Pyrazole-4-Carboxylic Acid Ethyl Ester	CID656969
10	2-[3-(2-Hydroxy-1,1-Dihydroxymethyl-Ethylamino)-Propylamino]-2-Hydroxymethyl-Propane-1,3-Diol	CID125132
11	6-(4-Difluoromethoxy-3-Methoxy-Phenyl)-2h-Pyridazin-3-One	CID5723
12	1-(4-Aminophenyl)-3,5-Dimethyl-1h-Pyrazole-4-Carboxylic Acid Ethyl Ester	CID656966
13	(S)-Rolipram	CID158758
14	Cilomilast	CID 151170
15	(R)-Rolipram	CID448055
16	3,5-Dimethyl-1h-Pyrazole-4-Carboxylic Acid Ethyl Ester	CID215436
17	1-(4-Methoxyphenyl)-3,5-Dimethyl-1h-Pyrazole-4-Carboxylic Acid Ethyl Ester	CID656965
18	AN2728	CID 44591583
19	Ibudilast	CID 3671
20	OPC-6535	CID 3025803
21	Apremilast	CID11561674
22	(4R)-4-(3-butoxy-4-methoxybenzyl)imidazolidin-2-one	CID6603746
23	3,5-DIMETHYL-1-PHENYL-1H-PYRAZOLE-4-CARBOXYLIC ACID ETHYL ESTER	CID656964
24	3-isobutyl-1-methyl-7H-xanthine	CID3758
25	4-[8-(3-nitrophenyl)-1,7-naphthyridin-6-yl]benzoic acid	CID 9999276
26	Urokinase	CID 9952109
27	Alteplase	CID 134222947
28	Reteplase	CID 134222312
29	Anistreplase	Appropriate structure not found
30	Tenecteplase	Appropriate structure not found
31	Streptokinase	CID9815560
32	Tranexamic Acid	CID5526
33	Aminocaproic Acid	CID 564
34	Benzamidine	CID 2332

35	Bicine	CID 8761
36	Desmoteplase	Appropriate structure not found
37	Aprotinin	CID 16130295
38	Cyclosporine	CID 5284373
39	L-Proline	CID 145742
40	(3r)-1-Acetyl-3-Methylpiperidine	CID449120
41	Ethyl Oxo(Piperidin-1-Yl)Acetate	Appropriate structure not found
42	(3s,6s,9r,10r,11s,12s,13e,15e,18s,21s)-18-{(1e,3e,7s,8s)-9-[(2s,3r,4s,5s,6r,9s,11s)-9-Ethyl-4-Hydroxy-3,5,11-Trimethyl-8-Oxo-1-Oxa-7-Azaspiro[5.5]Undec-2-Yl]-8-Hydroxy-1,7-Dimethylnona-1,3-Dienyl}-10,12-Dihydroxy-3-(3-Hydroxybenzyl)-6-Isopropyl-11-Methyl-	CID7890440
43	Palifermin	Appropriate structure not found
44	Pegaptanib	CID56603655
45	1-Hexadecylsulfonyl Fluoride	CID3572
46	Palmitic Acid	CID 985
47	Bosutinib	CID 5328940
48	Ponatinib	CID 24826799
49	Valproic Acid	CID 3121
50	Cyclosporine	CID 5284373
51	4-(5-benzo(1,3)dioxol-5-yl-4-pyridin-2-yl-1H-imidazol-2-yl)benzamide	CID 4521392
52	(6-(4-(2-piperidin-1-ylethoxy)phenyl))-3-pyridin-4-ylpyrazolo(1,5-a)pyrimidine	CID86346670
53	Aflatoxin B1	CID 186907
54	Copper Sulfate	CID24462
55	Tretinoin	CID 444795
56	Methyl Methanesulfonate	CID4156
57	Antirheumatic Agents	CID
58	trichostatin A	CID 444732
59	vorinostat	CID 5311
60	Acetaminophen	CID1983
61	Benzo(a)pyrene	CID 2336
62	Estradiol	CID 5757
63	Tetrachlorodibenzodioxin	CID 15625

Table S2. GO Database and KEGG database their corresponding P-values and genes for common differentially expressed genes

Category	Pathways	P-values	Gene ratio	Associated CDEGs
**Go biological process**	Cellular response to nutrient levels	0.00169	0.055	FOXO1, ITGB1, BECN1, FBXO22
	Regulation of endocytosis	0.00179	0.055	ITGB1, BCR, PPT1, AAK1
	Cellular response to extracellular stimulus	0.00311	0.055	FOXO1, ITGB1, BECN1, FBXO22
	Regulation of cell growth	0.0075	0.068	PPT1, SMARCA2, NRP1, ARHGEF11, EAF2
	Negative regulation of growth	0.00833	0.055	PPT1, SMARCA2, NRP1, EAF2
**Go molecular function**	Collagen binding	0.0152	0.027	CD44, ITGB1
	Adenylribonucleotide binding	0.0163	0.164	BCR, MKNK2, MYO6, NDUFA10 PDE4D, PRKCH, SMARCA2, AKT3, BCKDK, AAK1, ATAD2B, ABCC10
	Double_stranded RNA binding	0.0164	0.027	LRRFIP1, STAU2
	Phospholipid binding	0.0165	0.082	BCR, AKT3, GRB10, ARHGEF11 ASAP1, PHLDB2
	Adenyl nucleotide binding	0.0166	0.164	BCR, MKNK2, MYO6, NDUFA10 PDE4D, PRKCH, SMARCA2, AKT3, BCKDK, AAK1, ATAD2B, ABCC10
**Go cellular component**	Extrinsic to plasma membrane	0.00397	0.041	PLG, ST14, AAK1
	External side of plasma membrane	0.0066	0.055	PLG, CD44, ITGB1, GGTLC2
	Clathrin_coated vesicle	0.00911	0.055	AAK1, MYO6, PPT1, NECAP2
	Extrinsic to membrane	0.0133	0.041	PLG, ST14, AAK1
	Axon	0.0169	0.055	AAK1, MYO6, PPT1, NRP1
**KEGG**	Longevity regulating pathway - multiple species	0.00225	0.041	FOXO1, AKT3, EIF4EBP2
	Insulin signaling pathway	0.00258	0.055	FOXO1, AKT3, MKNK2, PPP1CB
	Fatty acid elongation	0.00579	0.027	HADHB, PPT1
	Inflammatory mediator regulation of TRP channels	0.00863	0.055	PPP1CB, PRKCH, IL1RAP
	Proteoglycans in cancer	0.01	0.055	AKT3, PPP1CB, CD44, ITGB1

Table S3: List of key TFs which are interacted with DEGs of Respiratory Diseases according to number of associated DEGs (≥18) in JASPAR database

TFs	No. of associated DEGs	Associated CDEGs
FOXC1	54	PLG, C14ORF93, PDE4D, AAK1, PPP1CB, ASAP1, MBNL3, MYADM, PHLDB2, SERPINB6, LRRFIP1, ASPHD2, EAF2, GRB10, PCBP2, ABCC10, MTFR1, SOX4, MYO6, CLIP4, FBXO22, XPO6, EDF1, SLC31A1, STAU2, CCDC82, ITGB1, ATXN7L1, TTC9, ANKRD44, PRMT2, TPM3, VPS53, BTBD9, NBPF1, AKT3, CD44, MBD4, RSBN1, ZNF213, ATAD2B, INVS, KCMF1, IL1RAP, BECN1, SMARCA2, EIF4EBP2, NDUFA10, CYB5B, CYP2C18, ECAP2, DIP2A, ARHGEF11, MKNK2
GATA2	33	PDE4D, PPP1CB, ASAP1, MBNL3, MYADM, PHLDB2, EAF2, GRB10, ABCC10, MTFR1, FBXO22, XPO6, EDF1, SLC31A1, STAU2, ITGB1, TPM3, VPS53, BTBD9, KCMF1, IL1RAP, SMARCA2.CYBMB, DIP2A, ARHGEF11, PHC3, ST14, PPIA, EIF3L, GGTLC2, PRKCH, ZNF512B
FOXL1	27	PLG, PDE4D, AAK1, PHLDB2, GLS, PCBP2, ABCC10, MTFR1, ST14, FBXO22, EDF1, SLC31A1, STAU2, TTC9, ANKRD44, BTBD9, NBPF1, INVS, KCMF1, IL1RAP, BECN1, SMARCA2, EIF4EBP2, CYB5B, NECAP2, MKNK2
YY1	25	PDE4D, PHLDB2, MTFR1, SLC31A1, BTBD9, INVS, KCMF1, EIF4EBP2, CYB5B, MBNL3, MYADM, EAF2, GRB10, SOX4, XPO6, CCDC82, ITGB1, ATXN7L1, VPS53, MBD4, RSBN1, ZNF213, CYP2C18, ARHGEF11, KDSR
POU2F2	22	PDE4D, AAK1, MBNL3, PHLDB2, SERPINB6, BCR, GRB10, GLS, PHC3, MTFR1, MYO6, FBXO22, XPO6, TTC9, PRMT2, AKT3, ATAD2B, EIF4EBP2, NDUFA10, CYP2C18, NECAP2, ARHGEF11
HINFP	18	PDE4D, MBNL3, MYADM, LRRFIP1, GRB10, MTFR1, XPO6, STAU2, ITGB1, ATXN7L1, TTC9, CD44, ZNF213, ATAD2B, SMARCA2, CYP2C18, MKNK2, ZNF512B

Table S4: List of key miRNAs which are interacted with DEGs of Respiratory Diseases according to number of associated DEGs (>10) in TarBase and miRTarBase

miRNAs	No. Of Associated DEGs	Associated CDEGs
hsa-mir-218-5p	13	PCBP2,TPM3,PHC3,CD44,NRP1,ITGB1,VPS53,PPIA,ATXN7L1,LRRFIP1,RSBN1,ERMP1,SERPINB6
hsa-mir-335-5p	13	BTBD9,FOXO1,PDE4D,NRP1,MKNK2,SOX4,GRB10,DIP2A,MYO6,GLS,ERMP1,ANKRD44,ST14
hsa-mir-16-5p	13	PDE4D,TPM3,MBD4,AKT3,TASP1,CLIP4,PHC3,CD44,KDSR,PPT1,NRP1,XPO6,PHLDB2
hsa-mir-106b-5p	11	PCBP2,CLIP4,MKNK2,ITGB1,SOX4,AAK1,TTC9,VPS53,PPIA,ASAP1,FBXO22
hsa-mir-15b-5p	11	FOXO1,PDE4D,TPM3,MBD4,AKT3,TASP1,CLIP4,PHC3,CD44, PPT1,PPP1CB

Table S5: List of key Chemicals which are interacted with DEGs of Respiratory diseases according to number of associated DEGs (>25) in Comparative Toxicogenomics database.

Chemicals	No. Of Associated DEGs	Associated CDEGs
Valproic Acid	55	BECN1, PDE4D, PPIA, PRKCH, C14ORF93, FOXO1, PHC3, NRP1, MKNK2, MYO6, NBPF1, PHLDB2, SOX4, GRB10, AKT3, BCR, ABCC10, ASAP1, ITGB1, GLS, CD44, PCBP2, MBD4, AAK1, ANKRD44, ASPHD2, ATXN7L1, CYB5B, EAF2, ERMP1, IL1RAP, KCMF1, KDSR, LRRFIP1, MBNL3, PRMT2, RSBN1, SMARCA2, ST14, STAU2, TASP1, TTC9, VPS53, EIF4EBP2, ATAD2B, CCDC82, MTFR1, MYADM, PPP1CB, XPO6, PPT1, ZNF512B, BCKDK, EIF3L, TPM3
Cyclosporine	45	CYP2C18, PPIA, C14ORF93, FOXO1, PHC3, CLIP4, NRP1, MKNK2, PHLDB2, SOX4, GRB10, AKT3, ABCC10, ASAP1, ITGB1, GLS, CD44, PLG, PCBP2, AAK1, ASPHD2, ATXN7L1, CYB5B, EAF2, ERMP1, KCMF1, KDSR, LRRFIP1, MBNL3, PRMT2, SMARCA2, STAU2, TTC9, EIF4EBP2, CCDC82, MTFR1, MYADM, NECAP2, XPO6, FBXO22, NDUFA10, PPT1, ZNF512B, EDF1, DIP2A
**Chemichal-1:** 4-(5-benzo(1, 3)dioxol-5-yl-4-pyridin-2-yl-1H-imidazol-2-yl)benzamide	39	PDE4D, PRKCH, C14ORF93, FOXO1, CLIP4, NRP1, NBPF1, PHLDB2, SOX4, AKT3, ASAP1, ITGB1, GLS, CD44, PCBP2, SLC31A1, MBD4, AAK1, ANKRD44, ASPHD2, ATXN7L1, CYB5B, EAF2, ERMP1, HADHB, IL1RAP, KCMF1, KDSR, LRRFIP1, MBNL3, PRMT2, RSBN1, SERPINB6, SMARCA2, ST14, STAU2, TASP1, TTC9, VPS53
**Chemichal-2:** (6-(4-(2-piperidin-1-ylethoxy)phenyl))-3-pyridin-4-ylpyrazolo(1, 5-a)pyrimidine	37	PDE4D, PRKCH, C14ORF93, FOXO1, CLIP4, NRP1, NBPF1, PHLDB2, SOX4, AKT3, ASAP1, GLS, CD44, PCBP2, SLC31A1, MBD4, AAK1, ANKRD44, ASPHD2, ATXN7L1, CYB5B, EAF2, ERMP1, IL1RAP, KCMF1, KDSR, LRRFIP1, MBNL3, PRMT2, RSBN1, SERPINB6, SMARCA2, ST14, STAU2, TASP1, TTC9, VPS53
Aflatoxin B1	36	CYP2C18, PDE4D, CLIP4, C14ORF93, NRP1, MYO6, PHLDB2, SOX4, GRB10, PLG, ASAP1, GLS, CD44, FBXO22, MBD4, AAK1, ANKRD44, ASPHD2, BTBD9, ATXN7L1, CYB5B, EAF2, IL1RAP, KCMF1, LRRFIP1, SLC31A1, GGTLC2, MBNL3, ST14, STAU2, ATAD2, MTFR1, CCDC82, MYADM, BCKDK, INVS
Copper Sulfate	35	PDE4D, PPIA, CLIP4, PLG, NDUFA10, FOXO1, PHC3, NRP1, MKNK2, PHLDB2, SOX4, GRB10, BCR, ASAP1, ITGB1, GLS, MBD4, AAK1, SLC31A1, CYB5B, ERMP1, IL1RAP, KCMF1, KDSR, LRRFIP1, MBNL3, PRMT2, SMARCA2, TTC9, EIF4EBP2, PPP1CB, TPM3, INVS, ZNF512B, BCKDK
Tretinoin	31	CYP2C18, PDE4D, PPIA, PRKCH, FOXO1, CLIP4, NRP1, MYO6, SOX4, GRB10, AKT3, ITGB1, GLS, CD44, SLC31A1, ANKRD44, ASPHD2, EAF2, ERMP1, KDSR, LRRFIP1, MBNL3,

		PRMT2, SERPINB6, SMARCA2, TTC9, EIF4EBP2, MTFR1, PPP1CB, XPO6, TPM3
Methyl Methanesulfonate	31	PPIA, PHC3, CLIP4, MYO6, NBPF1, SOX4, AKT3, ABCC10, ASAP1, ITGB1, GLS, CD44, SLC31A1, AAK1, ANKRD44, ASPHD2, CYB5B, EAF2, ERMP1, IL1RAP, KCMF1, LRRFIP1, RSBN1, STAU2, ATAD2B, CCDC82, NECAP2, PPP1CB, FBXO22, TPM3, DIP2A
Antirheumatic Agents	30	BECN1, FOXO1, PHC3, CLIP4, PHLDB2, GRB10, BCR, ITGB1, GLS, CD44, SLC31A1, AAK1, ANKRD44, ASPHD2, ATXN7L1, IL1RAP, LRRFIP1, SMARCA2, ST14, STAU2, BTBD9, CCDC82, MTFR1, NECAP2, XPO6, PPT1, ARHGEF11, EDF1,<br>EIF3L, TPM3
trichostatin A	29	PDE4D, PPIA, PRKCH, FOXO1, NRP1, NBPF1, PHLDB2, SOX4, AKT3, BCR, ABCC10, ASAP1, GLS, CD44, AAK1, ASPHD2, ATXN7L1, CYB5B, ERMP1, KCMF1, KDSR, LRRFIP1, MBNL3, RSBN1, SMARCA2, ST14, STAU2, CCDC82, TPM3
vorinostat	28	BECN1, PDE4D, PPIA, PRKCH, FOXO1, MKNK2, PHLDB2, SOX4, AKT3, BCR, ASAP1, ITGB1, GLS, CD44, PCBP2, AAK1, PLG, ATXN7L1, CYB5B, EAF2, IL1RAP, LRRFIP1, MBNL3, RSBN1, SMARCA2, ST14, ATAD2B, CCDC82
Acetaminophen	28	CYP2C18, PRKCH, PLG, FOXO1, PHC3, NRP1, MKNK2, MYO6, PHLDB2, GLS, CD44, PCBP2, MBD4, AAK1, ATXN7L1, CYB5B, KDSR, LRRFIP1, MBNL3, SMARCA2, MYADM, HADHB, FBXO22, NDUFA10, PPT1, SLC31A1, ZNF512B, BTBD9
Benzo(a)pyrene	28	PDE4D, PPIA, FOXO1, CLIP4, NRP1, MYO6, SOX4, GRB10, BCR, ASAP1, GLS, CD44, PLG, PCBP2, SLC31A1, ANKRD44, ATXN7L1, EAF2, MBNL3, SMARCA2, STAU2, TASP1, VPS53, ATAD2B, BTBD9, PPP1CB, FBXO22, INVS
Estradiol	26	BECN1, PPIA, FOXO1, PHC3, CLIP4, NRP1, MKNK2, NBPF1, SOX4, GRB10, BCR, ITGB1, GLS, CD44, PLG, SLC31A1, MBD4, ASPHD2, EAF2, MBNL3, ST14, STAU2, MYADM, XPO6, BCKDK, TPM3
Tetrachlorodibenzodioxin	26	CYP2C18, PDE4D, PRKCH, NRP1, PHLDB2, SOX4, BCR, ABCC10, ASAP1, ITGB1, GLS, CD44, PLG, SLC3A1, ATXN7L1, ERMP1, KDSR, LRRFIP1, SERPINB6, TTC9, ATAD2B, CCDC82, MTFR1, MYADM, XPO6, TPM3

Table S6: List of Repositioning Drug Candidates which are related with DEGs in Drug Bank database.

Drug Bank ID	Label	Mechanism of action	Drug Target Gene
DB00131	Adenosine monophosphate	Product of	PDE4D
DB00201	Caffeine		PDE4D
DB00651	Dyphylline	Inhibitor	PDE4D
DB00920	Ketotifen		PDE4D
DB01088	Iloprost	Inducer	PDE4D
DB01656	Roflumilast	Inhibitor	PDE4D
DB01791	Piclamilast	Not available	PDE4D
DB01954	Rolipram	Not available	PDE4D
DB01959	3,5-Dimethyl-1-(3-Nitrophenyl)-1h-Pyrazole-4-Carboxylic Acid Ethyl Ester	Not available	PDE4D
DB02676	2-[3-(2-Hydroxy-1,1-Dihydroxymethyl-Ethylamino)-Propylamino]-2-Hydroxymethyl-Propane-1,3-Diol	Not available	PDE4D
DB02918	6-(4-Difluoromethoxy-3-Methoxy-Phenyl)-2h-Pyridazin-3-One	Not available	PDE4D
DB03183	1-(4-Aminophenyl)-3,5-Dimethyl-1h-Pyrazole-4-Carboxylic Acid Ethyl Ester	Not available	PDE4D
DB03606	(S)-Rolipram	Not available	PDE4D
DB03849	Cilomilast	Inhibitor	PDE4D
DB04149	(R)-Rolipram	Not available	PDE4D
DB04271	3,5-Dimethyl-1h-Pyrazole-4-Carboxylic Acid Ethyl Ester	Not available	PDE4D
DB04469	1-(4-Methoxyphenyl)-3,5-Dimethyl-1h-Pyrazole-4-Carboxylic Acid Ethyl Ester	Not available	PDE4D
DB05219	AN2728	Inhibitor	PDE4D
DB05266	Ibudilast	Inhibitor	PDE4D
DB05298	OPC-6535	Not available	PDE4D
DB05676	Apremilast		PDE4D
DB06842	(4R)-4-(3-butoxy-4-methoxybenzyl)imidazolidin-2-one	Not available	PDE4D
DB07051	3,5-DIMETHYL-1-PHENYL-1H-PYRAZOLE-4-CARBOXYLIC ACID ETHYL ESTER	Not available	PDE4D
DB07954	3-isobutyl-1-methyl-7H-xanthine	Not available	PDE4D
DB08299	4-[8-(3-nitrophenyl)-1,7-naphthyridin-6-yl]benzoic acid	Not available	PDE4D

DB00013	Urokinase	Activator, Substance	PLG,ST14
DB00009	Alteplase	Activator	PLG
DB00015	Reteplase	Activator	PLG
DB00029	Anistreplase	Activator	PLG
DB00031	Tenecteplase	Activator	PLG
DB00086	Streptokinase	Activator	PLG
DB00302	Tranexamic Acid	Inhibitor	PLG
DB00513	Aminocaproic Acid	Inhibitor	PLG
DB03127	Benzamidine	Not available	ST14
DB03729	Bicine	Not available	PLG
DB04925	Desmoteplase	Not available	PLG
DB06692	Aprotinin	Not available	PLG
DB00091	Cyclosporine	Inhibitor Binder	P62937
DB00172	L-Proline	Binder	P62937
DB01742	(3r)-1-Acetyl-3-Methylpiperidine	Not available	P62937
DB02419	Ethyl Oxo(Piperidin-1-Yl)Acetate	Not available	P62937
DB03393	(3s,6s,9r,10r,11s,12s,13e,15e,18s,21s)-18-{(1e,3e,7s,8s)-9-[(2s,3r,4s,5s,6r,9s,11s)-9-Ethyl-4-Hydroxy-3,5,11-Trimethyl-8-Oxo-1-Oxa-7-Azaspiro[5.5]Undec-2-Yl]-8-Hydroxy-1,7-Dimethylnona-1,3-Dienyl}-10,12-Dihydroxy-3-(3-Hydroxybenzyl)-6-Isopropyl-11-Methyl-	Not available	P62937
DB00039	Palifermin	Not available	O14786
DB04895	Pegaptanib	Not available	O14786
DB02035	1-Hexadecylsulfonyl Fluoride	Not available	P50897
DBO3796	Palmitic Acid	Not available	P50897
DB06616	Bosutinib	Inhibitor	P11274
DB08901	Ponatinib	Inhibitor	P11274

Table S7. Proposed 11 potential drug complex selected based on docking results. Proposed 11 potential drug complex were carefully chosen based on their higher binding affinity scores. The fourth column displays the target protein's 3D structure along with potential therapeutic candidates. The neighboring residues (within 4 of the drug) are indicated in the fifth column of the 2D Schematic representation of the target protein with prospective medications interaction. The final column displays the important interacting amino acids.

Name of potential targets	Lead structure of compound	BA (kCal / mol)	3D Structure of Complex	2D Diagram of Interaction	Interacting amino acids
PDE4D	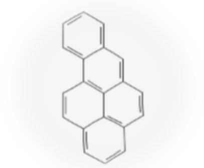 BENZO(a)PYRENE (CID2336)	-10.8	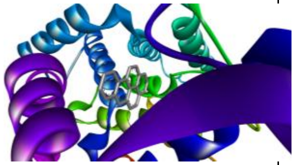	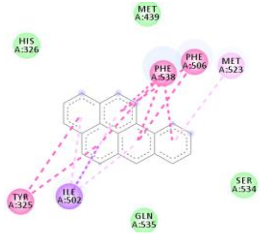	PHE538, PHE506, ET523, TYR325, ILE502, HIS326, MET439, GLN535, ER534
BCR	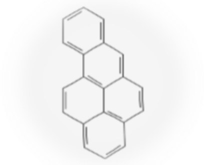 BENZO(a)PYRENE (CID2336)	-10	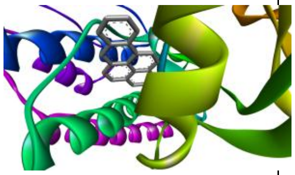	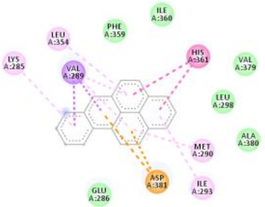	LYS285, LEU354, AL289, HIS361, MET290, ILE293, ASP381, PHE359, ILE360, VAL379, LEU298, LA380, GLU286
SMARCA2	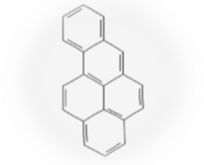 BENZO(a)PYRENE (CID2336)	-9.9	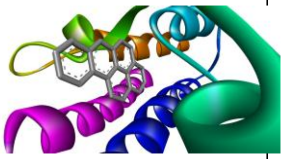	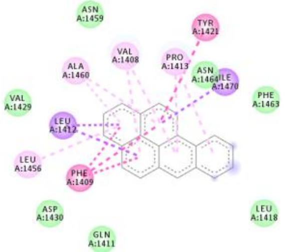	ALA1460, VAL1408, PRO1413, TYR1421, ASN1464, ILE1470, LEU1412, LEU1456, PHE1409, ASN1459, VAL1429, PHE1463, ASP1430, GLN1411, LEU1418
PPP1CB	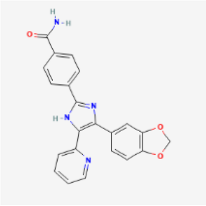 4-(4-(Benzo[d][1,3]dioxol-5-yl)-5-(pyridin-2-yl)-1H-imidazol-2-yl)benzamide (CID4521392)	-9.6	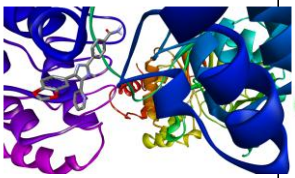	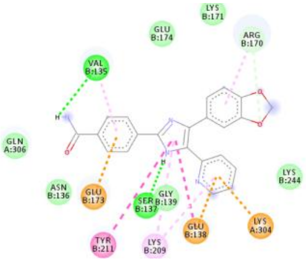	VAL135, GLU173, TYR211, SER137, GLY139, LYS209, GLU138, LYS304, ARG170, GLU174, LYS171, LYS244, ASN136, GLN306

HINFP	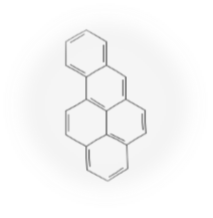 BENZO(a)PYRENE (CID2336)	-9.2	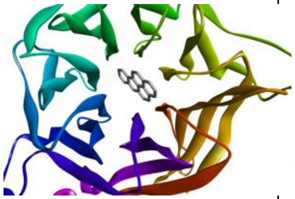	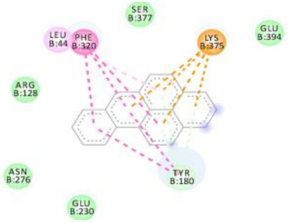	PHE320, LYS375, YR180, LEU44, SER377, GLU394, ARG128, ASN276, LU230
PCBP2	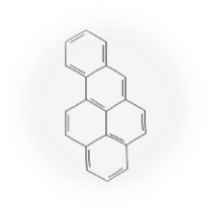 BENZO(a)PYRENE (CID2336)	-8.8	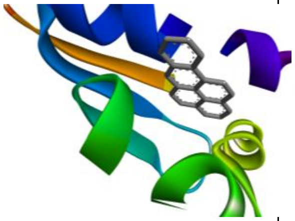	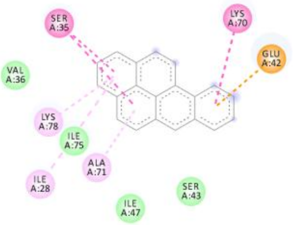	SER35, LYS70, GLU42, LYS78, ILE75, ILE28, ALA71, VAL36, ILE47, SER43
ATAD2B	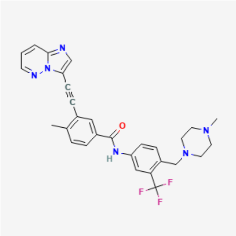 Ponatinib(CID24826799)	-8.5	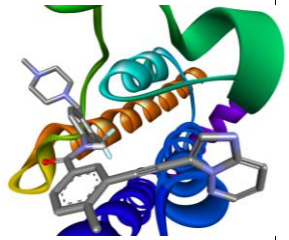	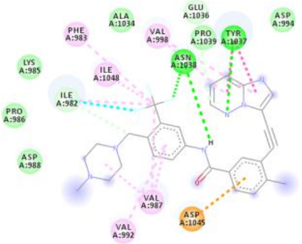	ILE982, ILE1048, PHE983, VAL998, ASN1038, PRO1039, TYR1037, GLU1036, VAL987, VAL992, ASP1045, ASP994, LYS985, ALA1034, PRO986, ASP988
YY1	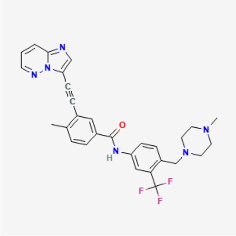 Ponatinib(CID24826799)	-8.3	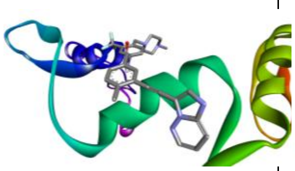	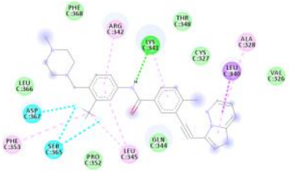	ARG342, LYS341, LEU340, ALA328, ASP367, PHE353, SER365, LEU345, GLN344, PRO352, LEU366, PHE368, THR348, CYS327, VAL326
CD44	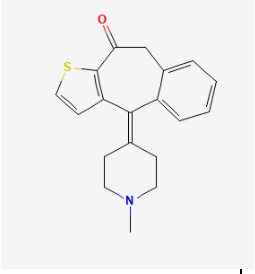 Ketotifen (CID3827)	-8.1	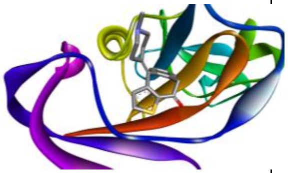	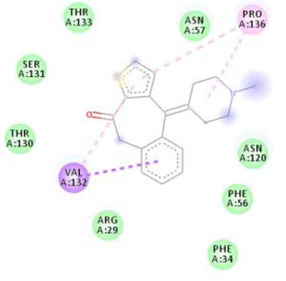	PRO136, VAL132, THR130, SER131, THR133, ASN57, ASN120, PHE56, PHE34, ARG29

ITGB1	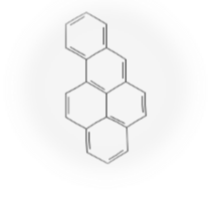 BENZO(a)PYRENE (CID2336)	-8.1	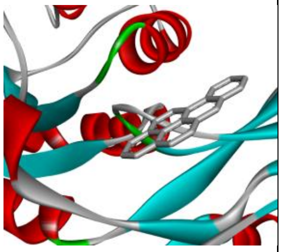	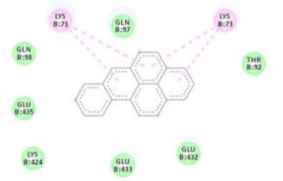	GLU432, GLU433, GLU435, GLN97, GLN98, THR92, LYS71, LYS73, LYS424
FOXC1	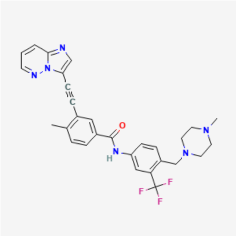 Ponatinib (CID24826799)	-8	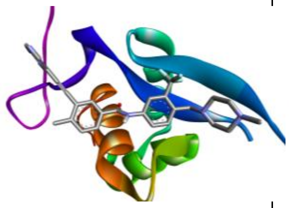	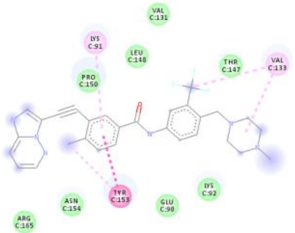	LYS91, PRO150, VAL133, THR147, TYR153, VAL131, LEU148, ARG165, ASN154, GLU90, LYS92
